# Impact of natural disaster on oral health: A scoping review

**DOI:** 10.1097/MD.0000000000033076

**Published:** 2023-02-22

**Authors:** Shinpei Matsuda, Hitoshi Yoshimura, Ichiro Kawachi

**Affiliations:** a Department of Dentistry and Oral Surgery, Unit of Sensory and Locomotor Medicine, Division of Medicine, Faculty of Medical Sciences, University of Fukui, Fukui, Japan; b Department of Social and Behavioral Sciences, Harvard T.H. Chan School of Public Health, Boston, MA, USA.

**Keywords:** disaster, earthquake, natural disaster, oral health, scoping review

## Abstract

Natural disasters may affect oral health as a result of serious damage to social function and public health. However, no article has systematically summarized the impact of natural disasters on oral health. This review aimed to map the existing literature on the impact of natural disasters on oral health. Targeted literature was searched using PubMed, Web of Science, Cochrane Library, and CINAHL databases with the keywords “disaster” and “oral health.” Eligibility criteria were established based on the Participant-Concept-Context model, and eligible studies were identified based on the Preferred Reporting Items for Systematic Reviews and Meta-Analyses 2020 flow diagram. Eight eligible studies related to earthquakes were included in this review. Of these, 7 studies were related to the 2011 Great East Japan Earthquake. Oral health status of victims of disaster was analyzed by assessing the following parameters: questionnaire surveys; examination for fungal infection; and analyses of the teeth, periodontal, and oral hygiene condition. Six studies suggested that natural disasters had a negative impact on oral health. Two studies could not determine the impact of natural disasters on oral health. Only 1 study analyzed the impact of disasters on oral health based on pre- and postdisaster surveys. This scoping review found that there was insufficient evidence to suggest a relationship between natural disasters and oral health and that there are biases in geographical areas and types of natural disasters in this research field. Further research is needed to promote evidence-based support by dental professionals during different disaster phases.

## 1. Introduction

The term “disaster” has been defined by the United Nations Office for Disaster Risk Reduction as “*a serious disruption of the functioning of a community or a society at any scale due to hazardous events interacting with conditions of exposure, vulnerability and capacity, leading to 1 or more of the following: human, material, economic and environmental losses and impacts*.”^[1]^ Disasters include “natural disasters,” such as earthquakes, tsunamis, typhoons, and floods, and “human disasters,” such as wars and economic disruptions. In the recent years, natural disasters such as storms, floods, and landslides related to climatic changes have been occurring in many parts of the world.^[2]^ At present, the coronavirus disease 2019 pandemic, caused by severe acute respiratory syndrome coronavirus 2, has had an economic impact globally and resulted in negative effects on health.^[3–5]^ The pandemic has exacerbated existing racial/ethnic and socioeconomic disparities in all aspects of health.^[6]^ Natural and human disasters can make it difficult to maintain physical and/or mental health and quality of life in the medical, social, and economic domains.^[7–9]^ Following the occurrence of a disaster, reducing health-related quality of life disparities and restoring social connections among community residents pose a challenging task.^[10,11]^ It is important to examine, adapt, and recover from these negative effects caused by various disasters through professional interventions and social support systems designed by politicians and experts in these fields.^[12,13]^

Natural disasters, such as earthquakes, tsunamis, typhoons, and floods, sometimes inflict enormous damage on houses, medical facilities, including dental clinics, and utility or power or economic infrastructures, such as water and electricity.^[1]^ People who experience lost or damaged their homes are forced to live outdoors or in public facilities. Such circumstances may lead to worsening of oral health due to the following factors: fear of water shortage and contamination, interruption of access to acute and preventative dental care, increased consumption of processed foods including sweets and snacks that could lead to the development of caries in children and adults, chronic stress resulting in inflammation and periodontitis, and worsened economic circumstances resulting in deteriorating self-care and tooth loss. Therefore, natural disasters may directly or indirectly affect oral health and oral oral health-related quality of life (OHRQoL) as a result of serious damage to social function and public health.^[14]^ Although its importance is obvious, no article has systematically summarized the impact of natural disasters on oral health. Thus, we sought to map and summarize the impact of natural disasters on oral health. This scoping review aimed to map the existing literature on the impact of natural disasters on oral health and discuss the mechanisms underlying these effects on oral health.

## 2. Methods

### 2.1. Study design

This scoping review was performed according to the Preferred Reporting Items for Systematic Reviews and Meta-Analyses extension for scoping reviews and adopted the Joanna Briggs Institute methodology framework (Supplemental Digital Content (Additional file 1, http://links.lww.com/MD/I537)).^[15–20]^ The protocol of this study has been registered in the Open Science Framework (https://osf.io/7xq3h/).

### 2.2. Information sources and literature search strategy

An electronic systematic literature search was conducted using PubMed, Web of Science, Cochrane Library, and CINAHL databases. The literature search strategy is presented in Table 1. The electronic searches were performed on July 30, 2021. Therefore, studies published before July 30, 2021, were included in this review.

**Table 1:** Electronic literature search strategy.

Database	Search strategy	Number of studies
PubMed	((disaster[MeSH Terms]) AND (oral health[MeSH Terms])) AND (English[Language])	24
Web of science	disaster (Topic) and oral health (Topic) and English (Language)	88
Cochrane library	disaster in Keyword AND oral health in Keyword	1
CINAHL	disaster AND oral health AND (English[Language])	24

### 2.3. Eligibility and exclusion criteria

Eligibility criteria were established based on the Participant-Concept-Context model outlined by the Joanna Briggs Institute guidelines as follows: **P:** all individuals; **C:** facing various natural disasters; and **C:** impact of various natural disasters on oral health.^[15–20]^

The exclusion criteria were as follows: reviews; conference papers or proceedings, letters to the editor, and commentaries; unavailable full studies; and studies in languages other than English.

### 2.4. Study selection process

Two independent reviewers, SM and HY, performed the study selection and review process. Based on the PRISMA 2020 flow diagram, the study selection process comprised the following steps: identification, screening, and included.^[21]^ Disagreements between reviewers were resolved through discussion and consensus. The reviewers (SM and HY) contacted the corresponding authors of the included studies if additional information was required. For this review, location of natural disaster, disaster type, timing of data collection, oral health assessment items, and impacts of natural disasters on oral health were extracted.

### 2.5. Critical appraisal

The reviewers performed critical quality appraisal of included articles using the mixed method appraisal tool version 2018.^[22,23]^

## 3. Results

### 3.1. Study selection process

**Figure 1.**
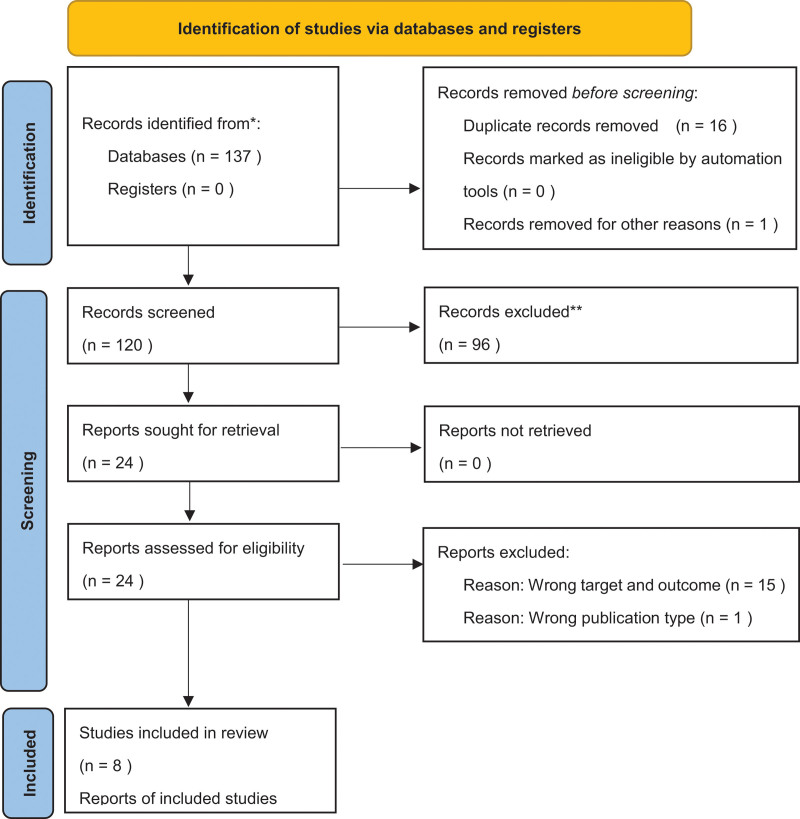
Flow diagram based on the Preferred Reporting Items for Systematic Reviews and Meta-Analyses 2020 flowchart template of the search and selection process.

Based on the electronic literature search, 137 studies were extracted (Fig. 1). One study published in Japanese was excluded, and 16 duplicate studies were removed. Furthermore, we excluded 96 studies after screening the titles and abstracts. Finally, after the assessment for eligibility was completed, 8 of 24 studies were included as shown in Table 2.^[14,24–30]^ The critical appraisal step was performed for each study.^[22,23]^

**Table 2:** Details of included studies in this review.

Authors	Publication yr	Area	Disaster	Target age (yrs)	Timing	Oral health assessment items	Impact	Relevant information
Hosokawa et al^[24]^	2012	Haiti, Japan	Earthquake	Children	10 mo after, immediately after	Teeth condition	Negative impacts	Place of residence
Sato et al^[25]^	2015	Japan	Earthquake	18≦	3 to 5 mo after	Questionnaire survey, teeth condition	Negative impacts	Place of residence, socioeconomic status, walking time, psychological status, status of dental clinic visit, problem of social connection
Kishi et al^[14]^	2015	Japan	Earthquake	18≦	9 mo after	Questionnaire survey, teeth and periodontal condition	Negative impacts	Evacuation from 1 home, socioeconomic status, systemic health status, psychological status, status of dental clinic visit
Tsuchiya et al^[26]^	2015	Japan	Earthquake	18≦	3 mo after to 2 years after	Questionnaire survey	Not applicable	Socioeconomic status, comorbid conditions, articles of taste, psychological status
Sato et al^[27]^	2017	Japan	Earthquake	60≦	3 years after	Questionnaire survey, oral *Candida* species examination, teeth, periodontal, and oral hygiene condition	No impacts	Relocation from 1 home, systemic diseases, medications, articles of taste
Matsuyama et al^[28]^	2017	Japan	Earthquake	65≦	7 mo before, 2 years after	Questionnaire survey, teeth condition	Negative impacts	Housing damage, comorbid conditions, socioeconomic status, education, loss of loved ones, psychological status
Rokaya et al^[29]^	2017	Nepal	Earthquake	18–70	5–8 mo after	Teeth, periodontal, and oral hygiene condition	Negative impacts	Systemic diseases, marital status, education, occupation, articles of taste
Tsuboi et al^[30]^	2020	Japan	Earthquake	20≦	2–9 yrs after	Questionnaire survey, teeth, periodontal, and oral hygiene condition	Negative impacts	Place of residence

### 3.2. Target area, disasters, and age

In this review, 7 of the 8 included studies were published by Japanese researchers and were related to the 2011 Great East Japan earthquake.^[14,24–28,30]^ All the included studies were related to earthquakes as natural disasters in Japan, Haiti, and Nepal.^[14,24–30]^ Three of the studies included subjects aged 18 years and older,^[14,25,26]^ and the remaining studies included participants of various ages.

### 3.3. Oral health assessment methods and timing

The 8 studies included in this review analyzed oral health status using the following assessment methods: questionnaire surveys; examination for fungal infection (*Candida* species); and analyses of the teeth and assessment of periodontal and oral hygiene condition.^[14,24–30]^ In 6 studies questionnaire surveys were performed to assess psychological status, lifestyle, medication, living condition, economic condition, and oral health behavior.^[14,25–28,30]^ Kishi et al used the General Oral Health Assessment Index scores to assess OHRQoL.^[14,31]^ Four of the 6 studies assessed psychological distress status.^[14,25,26,28]^ Seven studies assessed dental condition.^[14,24,25,27–30]^ Furthermore, periodontal assessment was performed in 4 studies.^[14,27,29,30]^ Sato et al investigated the loss of removable dentures related to the 2011 Great East Japan Earthquake.^[25]^ In addition, 1 study reported the prevalence of oral *Candida* species in elderly victims of natural disasters.^[27]^

A predisaster survey (7 months preceding the natural disaster) was performed in only 1 study.^[28]^ Postdisaster surveys were performed in all included studies.^[14,24–30]^ Regarding the timing of assessment, the included studies covered the first aid stage (less than 1 month after the disaster), defined as “immediately after the disaster occurred,” up to the medium-term rehabilitation stage (less than 1 year after disaster) and long-term rehabilitation stage (after 1 year from the disaster) were included.^[14,24–30,32]^

### 3.4. Impact of disasters on oral health

Six of the 8 studies suggested that disasters negatively impacted oral health.^[14,24,25,28–30]^ Only 1 paper consisted of pre- and postdisaster surveys, which analyzed the direct impacts of disasters on oral health.^[28]^ One study could not determine the impact of disasters on oral health.^[26]^ One study reported that *Candida* carriage status was not affected in postdisaster areas.^[27]^

### 3.5. Relevant information

All studies collected information on housing status, socioeconomic status, psychological status, systemic status and medications, marital status, status of dental clinic visit, education, occupation, and articles of taste.^[14,24–30]^ Systemic health status, including physical status and diseases, was assessed in 6 studies.^[14,25–29]^ Also, housing status, including evacuation from 1 home, relocation from 1 home, place of residence, and housing damage, was investigated in 6 studies.^[14,24,25,27,28,30]^ Socioeconomic status and psychological status were investigated in 4 studies.^[14,20,25,26]^ Articles of taste such as alcohol and cigarettes were investigated in 3 studies.^[26,27,29]^ Dental clinic visit status was reported by only 2 study.^[14,25]^

## 4. Discussion

This scoping review was performed to map the existing literature on the impact of natural disasters on oral health. This review clarified that research in this field has advanced over the last 10 years. All of the 8 studies in this review were related to earthquakes, and 7 were related to the 2011 Great East Japan Earthquake.^[14,24–30]^ Therefore, this review suggests that the 2011 Great East Japan Earthquake has advanced the field of research on the relationship between natural disasters and oral health.^[14,24–28,30]^ Furthermore, the assessment of oral health status associated with natural disasters was performed using the following methods: questionnaire surveys; examination for fungal infection; and analyses of the teeth, periodontal, and oral hygiene condition.^[14,24–30]^ Furthermore, in order to discuss the mechanisms mediating the effects of disasters on oral health, all of the included studies collected data associated with housing status, socioeconomic status, psychological status, systemic status and medications, marital status, status of dental clinic visits, education, occupation, and articles of taste.^[14,24–30]^

Although it is important to ascertain the oral health of natural disaster survivors before the disaster, e.g., by linking their information to dental health records preceding the disaster, only 1 study in our review utilized information predating the disaster.^[28]^ Hence there is insufficient evidence in the literature to elucidate clear mediating mechanisms linking disaster experiences to oral health conditions. There remain significant gaps in the range of participants, assessment items, geographical areas and types of natural disasters that have been studied. There is a need to develop consensus on the oral health assessment methods and timing of data collection as follows: before, during, and after disaster, including acute, intermediate, and long-term phases.^[32]^ These research results will lead to the advancement of evidence-based disaster support in each phase by dental professionals in the future.

Despite limited evidence, this review suggested that earthquakes, as natural disasters, tend to have a negative impact on oral health.^[14,24,25,28–30]^ However, in 2 of the reviewed studies, no association between disasters and oral health was found.^[26,27]^ Natural disasters are unpredictable and combined with the limited data currently available, it may be difficult to study and analyze their direct impact on oral health. Regular surveys on oral health by dental professionals may allow for a meaningful analysis of its relationship with these unpredictable disasters.

All of the studies included in this scoping review included relevant information for the analysis of the mechanisms mediating the impact of natural disaster on oral health.^[14,24–30]^ First, victims of natural disasters may face difficulties in securing clean water. The availability of water is critical especially during the acute phase after a natural disaster. Hosokawa et al reported that older people hesitated to wash their dentures because of insufficient water supply after the 2011 Great East Japan Earthquake, although no quantitative data on this issue has been reported by the authors.^[24]^

A second mechanism linking disasters to deterioration in oral health status is the potential for interruption of access to acute and preventive dental care. Dental clinic visits were reported by 2 studies based on a questionnaire survey during the acute phases following natural disaster, and both of these studies found evidence for interruption of dental treatment in the 2011 Great East Japan Earthquake.^[14,25]^ However, Kishi et al reported no difference in General Oral Health Assessment Index scores (an indicator of OHRQoL) between patients whose dental treatment had not been interrupted and those whose dental treatment had been interrupted but had resumed at the patient original dental clinic.^[14]^ Sato et al examined the negative impacts of disasters related to the loss of removable dentures, such as eating and speaking, and the problem of social connection and communication among residents.^[25]^ There was a significant difference in dental clinic visit status and in walking time between participants who had lost their dentures and those who had not.^[25]^ These studies could not suggest a direct link between natural disasters and interruption of access to dental care, and there were major differences in the research targets of these studies. Although there is currently insufficient evidence based on clear mediating mechanisms, dental professionals and health care providers should recognize the possibility of interruption of access to dental care of victims. Furthermore, it is important to encourage disaster victims to resume visits to the dental clinic for maintenance and improvement of oral health condition and to establish oral hygiene behaviors in the relatively acute phase after natural disaster. At the same time, it is necessary to ensure that lost removable dentures are replaced because dentures play an important role not only in direct oral function such as eating, but also in indirect oral function such as social activities and communication.^[25]^

A third pathway linking disasters to deterioration in oral health is increased consumption of processed foods including sweets and snacks, leading to the development of caries in children and adults. Housing and socioeconomic status may be relevant to this hypothesis; however, no direct quantitative data on this issue including diet and eating habit were collected in 7 of the studies included in this scoping review. Rokaya et al reported the daily number of meals.^[29]^ However, changes in dietary habits during the acute, intermediate, and long-term phases have not been reported in those studies. In addition, only 1 study gathered both pre- and postdisaster health-related information.^[28]^ Hence, we do not have sufficient information to assess the contribution of dietary changes to oral health status in the aftermath of disasters.

A fourth mechanism linking disasters to deterioration in oral health is via chronic stress and inflammation. Recent studies have suggested that chronic stress, including depression and anxiety, may accelerate the progression of periodontitis.^[33–36]^ Four of the studies included in this scoping review investigated periodontal condition or psychological status and only 1 study investigated both.^[14,25,26,28]^ Kishi et al found that serious psychological distress was significantly related to very poor OHRQoL.^[14]^ The relationship between mental health and oral health has been studied and reported in subjects of various ages, but it remains controversial.^[11,37,38]^ It is possible that chronic stress, for example, as a result of reduced socioeconomic status due to loss of employment and housing following the natural disaster, could affect the mental health of victims.^[39–43]^

The fifthly and last mechanism linking disasters to worse oral health is via deteriorating economic circumstances resulting in reduced self-care and tooth loss. Some studies reported that people with lower socioeconomic status had worse oral health than those with higher socioeconomic status.^[44–46]^ Socioeconomic status was investigated by 4 studies in this scoping review; only 1 study investigated the subjective deterioration of economic circumstances through comparison with economic status before the disaster.^[14,25,26,28]^ Kishi et al performed a cross-sectional study over nine months after natural disaster and reported that OHRQoL was more stongly associated with health assessment items than with socioeconomic aspects including house status and socioeconomic status.^[14]^ Based on a pre- and postdisaster survey, Matsuyama et al reported that a reduction in dental clinic visits among victims with worsened socioeconomic status may have contributed to tooth loss.^[28]^ In the remaining 2 studies, the direct relationship between socioeconomic status and oral health was not discussed.^[25,26]^ In the intermediate and long-term phases, changes in socioeconomic situation caused by natural disasters could have a significant impact on the affected people in many domains including systemic health, psychological and mental health, oral health, and status of dental clinic visits. Therefore, long-term and continuous cohort studies based on pre- and postnatural disaster surveys may play an important role in the development of policies for providing socioeconomic support to disaster victims.

This review has several limitations. First, studies using different oral health assessment methods were included in this review. Second, only 1 study included the results of a baseline survey in its analysis. Third, although we have discussed the potential mechanisms linking natural disasters to oral health status, few studies included in this scoping review could demonstrate these linkages.

Disasters, including human and natural disasters, occur worldwide, and may make it difficult to achieve some domains of the Sustainable Development Goals, which consist of 17 goals proposed by the United Nations.^[47]^ To achieve the sustainable development goals, dental professionals should also be involved in preparing, responding, and assisting in the recovery of victims of disasters.^[47]^ The number of humanitarian and oral health care programs has been growing worldwide, although the evidence supporting these interventions remains inadequate.^[48–50]^ Although natural disasters are unpredictable, there is a need to develop robust evidence for dental interventions that contribute to maintaining and improving oral health and OHRQoL during and after disasters based on further research.

## 5. Conclusion

This scoping review found that there was insufficient evidence to suggest a relationship between natural disasters and oral health. Furthermore, this review revealed several gaps in the research designs and coverage of different disasters across parts of the world. This study suggested that further research in various disasters and phases around the world is needed to clarify the mediating mechanisms of impact on oral health and promote evidence-based disaster support by dental professionals.

## Author contributions

**Conceptualization:** Shinpei Matsuda.

**Data curation:** Shinpei Matsuda.

**Formal analysis:** Shinpei Matsuda.

**Writing – original draft:** Shinpei Matsuda.

**Writing – review & editing:** Shinpei Matsuda, Hitoshi Yoshimura, Ichiro Kawachi.

## Supplementary Material


